# Versorgungsrealität patellastabilisierender Operationen: Ein Status quo unter aktiven Mitgliedern der Deutschen Gesellschaft für Orthopädie und Unfallchirurgie (DGOU)

**DOI:** 10.1007/s00132-022-04264-3

**Published:** 2022-05-23

**Authors:** Andreas Fuchs, Andreas Frodl, Tayfun Yilmaz, Matthias J. Feucht, Reinhard Hoffmann, Jörg Dickschas, Hagen Schmal, Kaywan Izadpanah

**Affiliations:** 1 Klinik für Orthopädie und Unfallchirurgie, Universitätsklinikum Freiburg, Hugstetter Str. 55, 79106 Freiburg, Deutschland; 2 Orthopädische Klinik Paulinenhilfe, Diakonieklinikum Stuttgart, Rosenbergstr. 38, 70176 Stuttgart, Deutschland; 3 Unfallchirurgie und Orthopädische Chirurgie, BG Unfallklinik Frankfurt am Main, Friedberger Landstr. 430, 60389 Frankfurt am Main, Deutschland; 4 Klinik für Orthopädie und Unfallchirurgie, Klinikum Bamberg, Buger Str. 80, 96049 Bamberg, Deutschland; 5 Department of Orthopedic Surgery, University Hospital Odense, Sdr. Boulevard 29, 5000 Odense C, Dänemark

**Keywords:** Gelenkinstabilität, Patella, Patellofemorales Gelenk, Präoperative Planung, Umfrage, Joint instability, Patella, Patellofemoral joint, Preoperative planning, Survey

## Abstract

**Hintergrund:**

Die patellofemorale Instabilität zählt zu den häufigsten Pathologien des Kniegelenks. Die Planung und Durchführung patellastabilisierender Operationen ist sehr variabel. Bezüglich der operativen Maßnahmen kommt der präoperativen Planung, gerade im Hinblick auf die häufig hohe Komplexität der zugrundeliegenden Pathologien, eine entscheidende Bedeutung zu.

**Fragestellung:**

Ziel dieser Studie war es, die aktuelle Versorgungsrealität in Bezug auf Planung und Durchführung patellastabilisierender Operationen unter Mitgliedern der Deutschen Gesellschaft für Orthopädie und Unfallchirurgie (DGOU) abzubilden. Des Weiteren sollte erhoben werden, ob ggf. automatisierte Analysen der zugrundeliegenden Anatomie die Planung und Durchführung patellastabilisierender Operationen (im Primär- und Revisionsfall) beeinflussen würden.

**Material und Methoden:**

Unter allen aktiven Mitgliedern der DGOU wurde per Mail eine anonymisierte Online-Umfrage mit 16 Fragen erhoben. 7974 Mitglieder wurden angeschrieben, 393 Rückmeldungen konnten anschließend analysiert werden.

**Ergebnisse:**

Die MPFL-Plastik (89,8 %) ist die am häufigsten durchgeführte Operation zur Patellastabilisierung. Dahinter folgen Tuberositasversatzoperationen (64,9 %), Korrekturosteotomien (51,2 %) und Trochleaplastiken (19,9 %). Die Wahl bezüglich des operativen Vorgehens fällt überwiegend auf Grundlage einer Kombination aus klinischen und radiologischen Befunden (90,3 %). Für die Entscheidung zur Operation werden hauptsächlich MRT-Bildgebung (81,2 %), Standard-Röntgenbilder (77,4 %) und Beinganzaufnahmen (76,6 %) herangezogen. Insgesamt würden 59,3 % der Befragten eine automatisierte Analyse für eine vereinfachte präoperative Planung und die Detektion von entscheidenden radiologischen Parametern (59,0 %) in Anspruch nehmen, sofern diese zur Verfügung stünden.

**Diskussion:**

Die Erhebungen dieser Umfrage unter Mitgliedern der DGOU weisen die MPFL-Plastik als zentralen Ansatzpunkt zur operativen Behandlung patellofemoraler Instabilitäten aus, diagnostisch ist die MRT-Bildgebung essenziell. Durch eine zukünftige Etablierung automatisierter Software-gestützter Analysemethoden könnte bei einer Vielzahl von Operateuren eine Erweiterung der radiologisch berücksichtigten Parameter in der Planung patellastabilisierender Operationen erreicht werden.

## Abstract (en)

**Background:**

Patellofemoral instability is one of the most common pathologies of the knee joint. The planning and implementation of patella-stabilizing operations are very variable. With regard to the operative measures, the preoperative planning is of decisive importance, especially with regard to the complexity of underlying pathologies.

**Objectives:**

The aim of this study was to depict the current healthcare reality in relation to planning and implementation of patella stabilizing operations in Germany. Furthermore, it was to be ascertained whether automated analysis options would facilitate the planning and implementation of surgical procedures (in primary and revision cases).

**Materials and methods:**

An online survey with 16 questions was collected by email among all active members of the German Society of Orthopedic and Trauma Surgery. 7974 members were surveyed; 393 responses could then be analyzed.

**Results:**

MPFL-reconstruction (89.8%) is the most frequently performed procedure, followed by tibial tubercle transfers (64.9%), osteotomies (51.2%), and trochleoplasty (19.9%). The choice of surgical approach is mainly based on a combination of clinical and radiological findings (90.3%). MRI imaging (81.2%), standard X‑ray images (77.4%), and full leg images (76.6%) are mainly used for operative treatment decisions. 59.3% of the respondents would appreciate better preoperative planning and 59.0% would implement more radiologically detectable parameters in their individual preoperative planning if these were automatically available.

**Conclusions:**

The findings of this survey among members of the DGOU identify the MPFL-reconstruction as the central operative approach in the treatment of patellofemoral instabilities, whereas MRI imaging is the diagnostic tool of choice. Future establishment of automated software-based analysis methods could allow a large number of surgeons to expand the radiological parameters taken into account when planning patella-stabilizing operations.

## Einleitung

Akute Patellaluxationen machen mit einer Inzidenz von ca. 7–49/100.000 2–3 % aller Kniegelenksverletzungen aus [1, 18, 23]. Aufgrund hoher Rezidivraten, patellofemoraler Knorpelverletzungen und Frakturen, sowie medialer Weichteilschädigungen können traumatische Patellaluxationen zu einer hohen Morbidität führen. Bis zu 50 % der Patienten nach Patellaerstluxation leiden dauerhaft unter Beschwerden, wie anteriorem Knieschmerz, verminderter Funktion sowie Patellofemoralarthrose [5, 12, 15, 21]. 17 % der Patienten nach erstmaliger und nahezu 50 % der Patienten nach einer Rezidiv-Patellaluxation erleben weitere Episoden einer patellofemoralen Instabilität [9]. Die häufig multifaktorielle Genese patellofemoraler Instabilitäten (PFI) einschließlich Insuffizienzen des medialen Retinakulums, Achsabweichungen, Rotationsfehlstellungen oder Trochleadysplasien ist mittlerweile bekannt [25]. Umso zentraler ist die Bedeutung einer möglichst genauen Analyse der zugrundeliegenden Pathologie vor einer etwaig durchzuführenden operativen Intervention. Ziel dieser Studie war es, die aktuelle Realität in Bezug auf Planung und Durchführung patellastabilisierender Operationen zu evaluieren und festzustellen, ob automatisierte Analysemöglichkeiten die Planung und Durchführung operativer Prozeduren erleichtern würden.

## Material und Methoden

**Figure 1:**
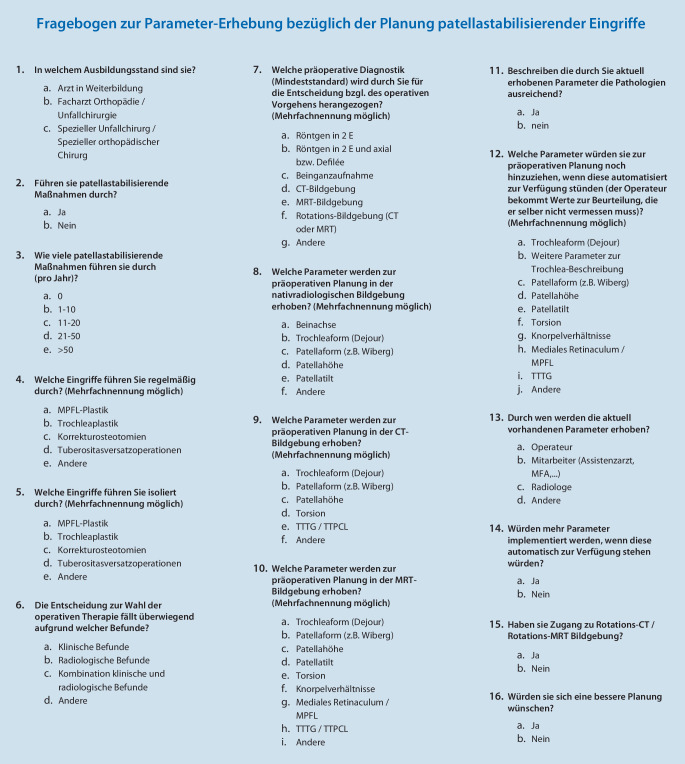

Es erfolgte eine Online-Umfrage (SurveyMonkey, San Mateo, CA, USA) unter allen Mitgliedern der Deutschen Gesellschaft für Orthopädie und Unfallchirurgie (DGOU) im Oktober 2020. Ein Umfragelink wurde per Mail an insgesamt 7974 Adressaten versendet, woraufhin ein Fragebogen für insgesamt 4 Wochen online zugänglich war und anonym beantwortet werden konnte. Der Fragebogen beinhaltete insgesamt 16 Fragen (Einfach-, und Mehrfachantworten), wobei sowohl eigene Erfahrung und aktuelle Planungs- und Durchführungsrealität als auch Verbesserungsmöglichkeiten in Bezug auf die präoperative Planung patellastabilisierender Operationen abgefragt wurden (Abb. 1). Die Erstellung des Fragebogens erfolgte in Zusammenarbeit mit den Vorsitzenden des Komitees „Patellofemoral“ der Deutschen Kniegesellschaft (DKG). Es konnten insgesamt 393 vollständig ausgefüllte Fragebögen in die Analyse integriert werden, dies entspricht einer Rücklaufquote von 4,9 %.

Die demographischen Daten wurden anhand deskriptiver Statistiken analysiert. Antworten auf Fragen der Online-Umfrage wurden anhand von Häufigkeiten in Prozent ausgewertet. Eine Berechnung der Stichprobengröße erfolgte nicht, da die Stichprobengröße der Anzahl der DGOU-Mitglieder entspricht und alle Mitglieder zur Teilnahme der Online-Befragung eingeladen wurden.

## Ergebnisse

### Demographische Analyse

393 Mitglieder (4,9 %) der DGOU beteiligten sich an der Umfrage, wobei der Fragebogen von allen Teilnehmern komplett beantwortet wurde. Unter den 393 Teilnehmern waren 4,6 % Ärzte in Weiterbildung und 31,8 % Fachärzte für Orthopädie und Unfallchirurgie, 63,6 % der Befragten führten die Qualifikation Spezieller Unfallchirurg bzw. Spezieller Orthopädischer Chirurg. 91,6 % der Befragten gaben an, patellastabilisierende Eingriffe durchzuführen, wobei hiervon 41,5 % 1–10 patellastabilisierende Eingriffe, 26,7 % 11–20, 17,1 % 21–50 und 7,9 % > 50 patellastabilisierende Operationen pro Jahr durchführen.

### Analyse der Planung und Durchführung patellastabilisierender Eingriffe

**Figure 2:**
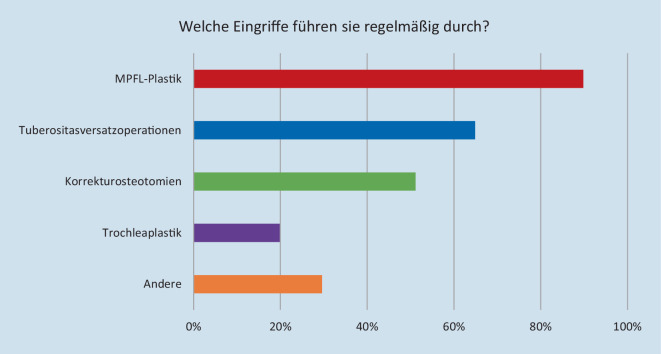

Bei der Frage, welche operativen Eingriffe bei Patienten mit patellofemoraler Instabilität (PFI) regelmäßig durchgeführt werden (Mehrfachnennung möglich), zeigte sich folgende Verteilung: MPFL-Plastik 353 (89,8 %), Tuberositasversatzoperation 255 (64,9 %), Korrekturosteotomie 201 (51,2 %), Trochleaplastik 78 (19,9 %), andere 81 (20,6 %). Auch bei isoliert durchgeführten Operationen zeigt die MPFL-Plastik die größte Verbreitung (87,5 %), gefolgt von Tuberositasversatzoperationen (43,3 %), Korrekturosteotomien (39,9 %) und Trochleaplastiken (8,4 %) (Abb. 2).

Es gaben 90,3 % der Befragten an, dass die Entscheidung bzgl. der operativen Therapie aufgrund einer Kombination aus klinischen und radiologischen Befunden getroffen wird. Bei 6,9 % erfolgt die Therapieentscheidung überwiegend aufgrund klinischer, bei 2,8 % auf Basis radiologischer Befunde.

**Figure 3:**
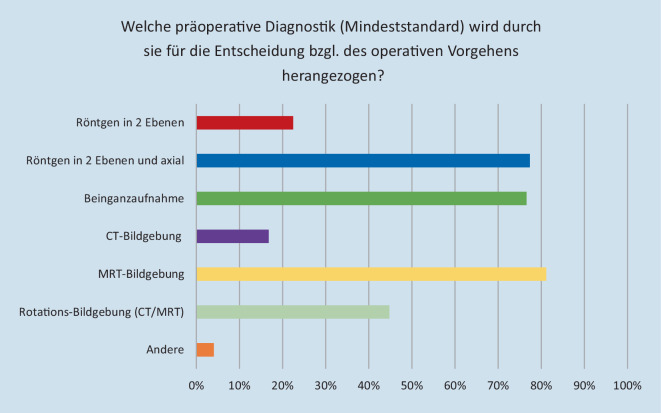

Als Mindeststandard für die Entscheidung bzgl. des operativen Vorgehens (Mehrfachnennung möglich) wurde die MRT-Bildgebung (81,2 %) am häufigsten genannt. Die Verteilung der weiteren Antworten zeigte sich wie folgt: Röntgen in 2 Ebenen und axial (77,4 %), Röntgen Beinganzaufnahme (76,6 %), Rotationsbildgebung (CT oder MRT) (44,8 %), Röntgen in 2 Ebenen (26,5 %), CT-Bildgebung (16,8 %), andere (4,1 %) (Abb. 3).

In der präoperativen nativradiologischen Diagnostik (Mehrfachnennung möglich) werden von den Teilnehmern der Umfrage Beinachse (91,6 %), Trochleaform (83,5 %), Patellahöhe (80,4 %), Patellaform (71,5 %) und Patella-Tilt (68,9 %) erhoben. Im Fall einer präoperativ durchgeführten CT-Diagnostik wird durch die Teilnehmer am Häufigsten der TTTG („tibial tuberosity – trochlear groove“)/TTPCL („tibial tuberosity – posterior cruciate ligament“) – Abstand erhoben (75,8 %), weitere erhobene Parameter sind: Trochleaform (68,5 %), Torsion (59,0 %), Patellaform (53,7 %) und Patellahöhe (45,0 %). Die für die präoperative Planung relevanten Parameter in der MRT-Bildgebung waren Knorpelverhältnisse (92,4 %), mediales Retinakulum/MPFL (88,6 %), Trochleaform (75,6 %), TTTG/TTPCL (72,8 %), Patellaform (57,8 %), Patellahöhe (47,3 %), Patella-Tilt (46,8 %) und Torsion (36,1 %).

Es gaben 356 Teilnehmer (90,6 %) an, dass die für die operative Entscheidung relevanten Parameter durch den Operateur erhoben werden, 25 Befragte (6,4 %) nannten Radiologen, 10 Befragte (2,6 %) andere Mitarbeiter (Assistenzärzte, Medizinische Fachangestellte, ...).

### Verbesserungspotenzial durch automatisiert verfügbare Parameter

Es gaben 262 Befragte (66,7 %) an, dass die durch sie aktuell erhobenen Parameter die jeweilige Pathologie vollständig beschreiben würden, 131 Teilnehmer (33,3 %) beantworteten dies Frage hingegen mit „nein“.

**Figure 4:**
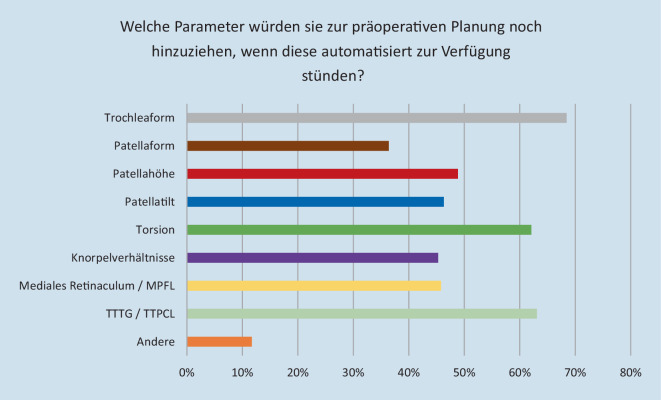

Falls den Teilnehmern automatisiert weitere Parameter zur Verfügung stünden, würden diese mehrheitlich die Trochleaform (68,5 %) in die präoperative Planung implementieren. Weitere erhobene Parameter im Falle einer automatisierten Erhebung sind TTTG/TTPCL (63,1 %), Torsion (62,1 %), Patellahöhe (48,9 %), Patella-Tilt (46,3 %), mediales Retinakulum/MPFL (45,8 %), Knorpelverhältnisse (45,3°) und Patellaform (36,4 %) (Abb. 4).

Von den Befragten gaben 59,0 % an, mehr Parameter in der Planung patellastabilisierender Eingriffe zu betrachten, wenn diese automatisiert zur Verfügung stehen würden. 41,0 % würden auch in diesem Fall keine weiteren Parameter in die Entscheidungsfindung implementieren.

Eine bessere Planung patellastabilisierender Operationen wünschen sich 59,3 % der Befragten, 40,7 % sind mit ihrer aktuellen Planung zufrieden.

## Diskussion

Die wichtigsten Erkenntnisse der durchgeführten Umfrage unter Mitgliedern der Deutschen Gesellschaft für Orthopädie und Unfallchirurgie sind:

- Die MPFL-Plastik ist die am Häufigsten durchgeführte operative Intervention zur Patellastabilisierung unter Mitgliedern der DGOU, sowohl in Kombination mit anderen patellastabilisierenden Verfahren als auch isoliert.
- Diagnostisch ist die MRT-Bildgebung essenziell.
- 59,3 % der Befragten würden sich in Bezug auf die radiologische Bildgebung eine bessere Planung patellastabilisierender Operationen wünschen und 59,0 % würden mehr radiologische Parameter in die präoperative Planung integrieren, wenn diese automatisiert zur Verfügung stehen würden.

Obwohl die patellofemorale Instabilität (PFI) zu den relevantesten Pathologien im Bereich des Kniegelenks zählt, bleibt die Behandlung von Patienten mit PFI weiterhin komplex und teilweise heterogen. Das Verständnis bezüglich Ätiologie und richtiger Therapiestrategie wird durch eine heterogene Patientenpopulationen, eine Vielzahl von Operationstechniken und den Mangel an langfristigen, hochqualifizierten klinischen Ergebnisstudien erschwert [14].

Ein Hauptaugenmerk der durchgeführten Befragung war die Abbildung der aktuellen Versorgungsrealität unter den Mitgliedern der DGOU. Hierbei zeigte sich, dass die MPFL-Plastik sowohl insgesamt (89,9 %) als auch bei isoliert durchgeführten Eingriffen (87,5 %) die weiteste Verbreitung findet. Aufgrund der Tatsache, dass die MPFL-Plastik gute klinische Ergebnisse zeigt und es sich dabei um eine im Vergleich weniger invasive, kostengünstige und risikoarme operative Maßnahme zur Patellastabilisierung handelt, ist es nicht verwunderlich, dass diese Methode durch die meisten Befragten bevorzugt wird [11, 20]. Hier sollte jedoch kritisch ergänzt werden, dass eine alleinige MPFL-Plastik, gerade im Hinblick auf Fehleranalysen in Revisionseingriffen, nicht sämtliche Ursachen einer patellofemoralen Instabilität adäquat adressiert [8]. Tuberositasversatzoperationen (64,9 %) und Korrekturosteotomien (51,2 %) zeigen ebenfalls eine relativ weite Verbreitung, wohingegen Trochleaplastiken mit 19,9 % im Vergleich selten durchgeführt werden. Die Trochleaplastik ist eine etablierte Operationsmethode, welche darauf abzielt, eine physiologische Anatomie der Trochlearille herzustellen [3]. Unterschiedliche Techniken und Kombinationseingriffe, mit dem Ziel einer möglichst optimalen Wirksamkeit zur Behandlung der PFI bei Patienten mit hochgradiger Trochleadysplasie, wurden veröffentlicht [2, 4, 6, 16, 17]. Auch in anderen Studien zur Verbreitung unterschiedlicher Operationstechniken in der Behandlung der PFI zeigte sich eine ähnliche Verbreitung der Trochleaplastik, wobei die Durchführung dieser Methode bei Operateuren mit mehr Erfahrung verbreiteter ist [11, 14]. Geierlehner et al. berichteten in einer Umfrage unter Mitgliedern der Gesellschaft für Arthroskopie und Gelenkchirurgie (AGA), dass lediglich 25 % aller Befragten, jedoch 60 % sog. „High-volume surgeons“-Trochleaplastiken durchführen [11]. Liu et al. berichteten gar von einem Anteil von 80 % der Befragten im Level der „absolute experts“, die Erfahrungen mit Trochleaplastiken aufwiesen [14]. Diese Ergebnisse zeigen, dass die Trochleaplastik als technisch anspruchsvoller chirurgischer Eingriff angesehen wird, welcher hauptsächlich von erfahrenen Chirurgen mit großen Fallzahlen durchgeführt wird. Zwar gaben 91,6 % der im Rahmen dieser Studie befragten Teilnehmer an, patellastabilisierende Eingriffe durchzuführen, eine Mehrheit jedoch in vergleichsweiser geringer Fallzahl von 1–10 (41,5 %) bzw. 11–20 (26,7 %) pro Jahr. Dies bestätigt die bereits durch o. g. Studien beschriebene Verbreitung der operativen Maßnahmen in Abhängigkeit von den Fallzahlen.

Hinsichtlich des Mindeststandards bei der präoperativen radiologischen Diagnostik gaben die meisten der Befragten (81,2 %) die MRT-Bildgebung an. Die herausragende Bedeutung der MRT-Bildgebung im Kontext patellofemoraler Pathologien zeigt sich neben den Ergebnissen im Rahmen dieser Umfrage auch anhand unterschiedlicher Publikationen [7, 13, 14, 19, 22, 24]. Nicht nur die exakte Analyse der vorliegenden Anatomie, sondern auch die Detektion von Begleitverletzungen im Rahmen stattgehabter Luxationsereignisse ist hier von entscheidender Bedeutung. Auch die Mehrheit der Befragten in der Studie von Liu et al. empfahl die Durchführung einer MRT zur Detektion freier Gelenkkörper und osteochondraler Läsionen bei Patienten mit erstmaligem Luxationsereignis [14]. Diese Ergebnisse stehen im Einklang mit früheren Studien, die über Knorpelschädigungen bei 39–96 % der Patienten mit Patellaluxationen berichteten [7, 13, 19, 22, 24].

Zusätzlich zur Erfassung der präoperativen Diagnostik und aktuellen Behandlungsrealität lag ein wichtiger Fokus dieser Befragung auf der Untersuchung möglicher Verbesserungspotenziale durch automatisiert verfügbare Parameter. Durch die zunehmende Implementierung softwaregestützter Analysemethoden zeigt sich bereits jetzt eine Verbesserung der Analysemöglichkeiten vorliegender Pathologien, beispielsweise durch die 3‑D-Darstellung anatomischer Strukturen im Kniegelenk [10]. Zukünftig soll dieser Fortschritt zu einer automatisierten, MRT-basierten Analyse der vorliegenden patienteneigenen Kniegelenksanatomie mit Darstellung der relevanten Parameter ausgebaut werden. Daten bzgl. der Trochleaform, Patellahöhe/-Tilt, Knorpelverhältnisse sowie Parameter wie TTTG oder TTPCL würden so automatisiert anhand routinemäßig durchgeführter MRT-Aufnahmen zur Verfügung gestellt werden.

Insbesondere Parameter wie Trochleaform und TTTG/TTPCL würden im Falle einer automatisierten Erhebung von den Befragten dieser Studie in die präoperative Planung implementiert werden. Diese Erkenntnis, sowie der Anteil von 59,0 % der Befragten, welche im Falle einer automatisierten Erhebung mehr Parameter in der Operationsplanung betrachten würden, zeigen das Potenzial solcher Software-gestützter Analyseverfahren in der Behandlung patellofemoraler Pathologien. Vor dem Hintergrund, dass sich 59,0 % der im Rahmen dieser Studie befragten Chirurgen eine bessere Planung patellastabilisierender Operationen wünschen würden, ist die Weiterentwicklung dieser Analysemethoden ein vielversprechender Ansatz, welcher auf Basis bereits validierter Software-Modelle verfolgt wird. Somit kann potenziell durch eine solche Verbesserung der Diagnostik mittels automatisierter Software-gestützter Analysemethoden, wie bereits in ähnlicher Weise z. B. in der Planung endoprothetischer Operationen, eine Verbesserung der Patientenversorgung gerade bei Operateuren mit geringeren Fallzahlen erreicht werden.

## Fazit für die Praxis

- Die MPFL(mediales patellofemorales Ligament)-Plastik ist die am Häufigsten durchgeführte operative Intervention zur Patellastabilisierung unter Mitgliedern der DGOU (Deutsche Gesellschaft für Orthopädie und Unfallchirurgie), sowohl in Kombination mit anderen patellastabilisierenden Verfahren als auch isoliert. Diagnostisch ist die MRT-Bildgebung essenziell.
- 59,3 % der Befragten würden sich in Bezug auf die radiologische Bildgebung eine bessere Planung patellastabilisierender Operationen wünschen und 59,0 % würden mehr radiologische Parameter in die präoperative Planung integrieren, wenn diese automatisiert zur Verfügung stehen würden.
- Durch eine zukünftige Etablierung automatisierter Software-gestützter Analysemethoden könnte bei einer Vielzahl von Operateuren eine Erweiterung der radiologisch berücksichtigten Parameter in der Planung patellastabilisierender Operationen erreicht werden.

## Abkürzungen

DGOU: Deutsche Gesellschaft für Orthopädie und Unfallchirurgie
DKG: Deutsche Kniegesellschaft
MPFL: Mediales patellofemorales Ligament
PFI: Patellofemorale Instabilität
TTPCL: Tibial tuberosity – posterior cruciate ligament
TTTG: Tibial tuberosity – trochlear groove
